# Coroners

**Published:** 1880-01

**Authors:** 


### CORONERS.

The importance of the position of Coroner, we are led to believe, is not properly appreciated by the public, else would we not see it held by the very incompetent men that usually obtain possession of the office. The Coroner should be a man of undoubted ability as a physician—a man past the middle age of life, in possession of a large and lucrative practice, and of unimpeachable integrity. Such a physician would be able to determine, without a moment's hesitation, whether a Coroner's inquest is necessary or not, when summoned to the scene of a sudden, mysterious or tragic death. He would not empannel a jury, summon a long list of witnesses, and spread his examination over several days, postponing it from time to time that the largest fees might accrue to his ingenious method of examination, but would terminate the proceedings as promptly as possible and save the people from unnecessary expense.

An intelligent Coroner, one worthy the name, would dismiss more than half the calls for inquests as utterly without need of expensive interference.

But it has come to pass that the office is made a sort of asylum for impecunious and incapable young men, just out of their collegiate swaddling clothes, who seek the office not for the honor it confers, not with the laudable desire to serve the community faithfully and well, but for the vastly more important consideration, to them, of obtaining a livelihood while they are waiting for the time when a supporting remuneration may come to them through their regular and legitimate practice. These youngsters, with an eye upon the emoluments of the office, importune their friends, plead with the older physicians, enlisting their sympathy, organize a system of caucus cramming, and finally succeed in having themselves nominated for one of the most important offices in the county, and one for which they are neither professionally nor morally capable of filling. Having abundance of time, they devote all their energies to an election ; and, as the office is one for which the community pay little heed, they succeed in obtaining the prize and forthwith lay their plans to make the office "pay" as largely as possible. An inquest is held upon the slightest provocation, and a post-mortem examination also, even where not a shadow of doubt exists as to the cause of death. But the inquest, the witnesses sworn, and the post-mortem examination are all charged for in the bill, so bringing the ambitious and diligent Coroner a handsome income for his meddlesome and officious interference. Of late years, he has added a new means of extracting money from the public treasury in instituting fire inquests. Should a fire occur, the origin of which is shielded in the slightest mystery, the Coroner has it within his power to institute an inquest to determine the cause of the fire, although the community nor the proprietor may not derive the slightest benefit from discovering the cause. But a bill is duly rendered at the end of the year, and another considerable item added to the county's indebtedness.

It is the poorest sort of economy to make coroners of young physicians who *need* the fees of such an office for their support. It is too much of a temptation to them to pursue the duties (?) of their office too diligently. They are apt to find too many mysterious deaths, which inclination, if not speedily checked, may extend to making subjects for a coroner's inquest of us all, a contemplation that must add increased fear of death in the mind of every county Supervisor, if not in that of the good people in general.

We protest that the great majority of inquests are unnecessary. That no intelligent and honest coroner would institute them. That a reform is necessary in this particular. That the remedy is to elect the very best physician that can be persuaded to take the office.

